# Prevalence of Self-medication Practices for Oral Health Problems among Dental Patients in a dental college: A Descriptive Cross-sectional Study

**DOI:** 10.31729/jnma.4866

**Published:** 2020-04-30

**Authors:** Rosina Bhattarai, Sunita Khanal, Sujita Shrestha

**Affiliations:** 1 Department of Community Dentistry, College of Medical Sciences, Bharatpur, Chitwan, Nepal; 2 Department of Community Dentistry, Kantipur Dental College, Dhapasi, Kathmandu, Nepal

**Keywords:** *oral health*, *prescription drugs*, *self medication*

## Abstract

**Introduction:**

Self-medication means the use of medications for the treatment of any disease on their own, without consulting any healthcare professional. At times self-medication can be useful if practiced correctly by saving time and money, whereas disadvantages often occur due to lack of evaluation by trained medical professionals and delay ineffective treatment and can result in unnecessary expenses and drug dependence. This study was conducted to find out the self-medication behavior and its associated factors among patients visiting a dental hospital in Kathmandu.

**Methods:**

A hospital-based, cross-sectional study was conducted on 265 patients in Kantipur Dental College from December 2019 to January 2020 among the patients attending the dental Out Patient Department. Ethical clearance was obtained from the Institutional Review Committee of Kantipur Dental College. A convenience sampling technique was used. Proformas were prepared in English, translated to Nepali and re-translated to English by the back-translation method. Data entry was done in Microsoft Excel and analysis in SPSS 20. Descriptive statistics was done.

**Results:**

The prevalence of self-medication practice was found to be 166 (62.6%). Out of total participants, 99 (59.6%) consumed medicines for few days only and the most common triggering factor was found to be toothache in 101 (60.8%) participants. The most common reason for self-medication was found to be a previous experience of treating similar illnesses.

**Conclusions:**

The prevalence of self-medication was found to be low as compared to the study done in similar settings. Self-medication practice is a sensitive issue that hasn't been given the required consideration. It is of concern to find every other dental patient being indulged in self-medication whether in the present or the past.

## INTRODUCTION

Self-medication practice means the use of medicines without prescription given by doctors for treating self-recognized conditions. It is an important element of self-care alongside hygiene, nutrition, lifestyle, environmental and socioeconomic factors.^1^ Advantages include saving cost and time while disadvantages include wasting resources, misdiagnosis, drug resistance and interactions etc.^2,3^

In Nepal, drug-stores often are people's first contact point with the healthcare system.^4^ Drugs these days have become more accessible to people.^5^ Many “prescription-only” drugs can be bought over-the-counter (OTC) in developing countries like Nepal.^6^ The most commonly used drug for self-medication worldwide was found to be antibiotics which are also commonly used in dentistry, therefore, necessitating the need for this study.^7^

Many studies have been conducted on self-medication for general illness. However, no studies have been done for dental ailments in Nepal. The present study was conducted to assess the prevalence of self-medication and among patients visiting a dental hospital in Kathmandu.

## METHODS

A hospital-based, descriptive cross-sectional study was conducted in Kantipur Dental College, Teaching Hospital and Research Centre among the patients attending the out-patient department. Convenience sampling technique was used and the required sample size was calculated by using the following formula:

n = Z^2^ X p X q/e^2^ where

Where,

n = sample size
p = prevalence was of self-medication was 81.35% from the study by Banerjee et al.^8^
q = 1-p i.e. 18.65%
e = margin of error i.e. 5%

A non-response rate of 10% was added and the final sample size was calculated to be 265.

Data collection was done from December 2019 to February 2020. Inclusion criteria included adult patients greater than 18 years of age and those who gave consent for the study. Exclusion criteria included those working in the field of healthcare, those who did not give consent for the study and those who submitted incomplete proformas. Ethical clearance was obtained from the Institutional Review Committee of Kantipur Dental College, Teaching Hospital and Research Centre. The objective of the study was explained and consent was taken from the participants before the study.

The questionnaire was developed by using the study by Komal Raj et al.^9^ and it was validated among 30 patients visiting the hospital. The first section of the questionnaire contained information on the demographic details of the participants. The second section included distance of the respondent's house to the nearest health facility. The third section included questions related to knowledge about self-medication related to oral health problems, triggering factors, reason, the response of people, the source of buying and measures that are taken if the problem persists. The survey proforma was prepared in English which was translated to the Nepali language and re-translated to English by a back-translation method by the faculties of the institute. Data entry was done in Microsoft Excel and analysis was done in SPSS Version 20 software. Descriptive statistics was done where suitable.

## RESULTS

The total participants of the study were 265 with a mean age of 30.46 years. The majority of the participants belonged to the age group of 18-24 years i.e. 110 (41.5%) and 146 (55.1%) of the participants were females while 119 (44.9%) were males (Table 1).

**Table 1:** Socio-demographic factors of study participants.

Characteristics	Frequencyn (%)
Gender	
Male	119 (44.9)
Female	146 (55.1)
Age group	
18-24 years	110 (41.5)
25-34 years	84 (31.7)
35-44 years	28 (10.6)
45-54 years	30 (11.3)
>55 years	13 (4.9)
Marital status	
Married	144 (54.3)
Unmarried	121 (45.7)
Education	
Illiterate	15 (5.7)
Up to secondary level	42 (15.8)
Higher secondary level	89 (33.6)
Bachelors	99 (37.4)
Masters	20 (7.5)

The distance between the homes of the majority of the participants and the nearest health center was less than 1km (Table 2).

**Table 2:** Distance from the nearest health center.

Distance	Frequency n (%)
<1 km	152 (57.4)
1-2 km	74 (27.9)
>2 km	39 (14.5)

Out of the total 165 participants, the prevalence of self-medication practice among participants was found to be 62.6%.

The duration of self-medication usage by 99 (59.6%) participants was for few days, triggering factors for the majority of them i.e. 101 (60.8%) being toothache. Of the total participants, 101 (60.8%) of them had previous experience in treating similar illnesses (Table 3).

**Table 3:** Duration, triggering factors and reasons for self-medication.

Questions	Frequency n (%)
Duration of self-medication usage	
Few days	99 (59.6)
Few weeks	16 (9.6)
Till condition subsides	51 (30.7)
Triggering factor	
Toothache	101 (60.8)
Gum bleeding	18 (10.8)
Bad breath	12 (7.2)
Swelling	10 (6.0)
Others	20 (15.1)
Reason for self-medication	
Lack of time	46 (27.7)
Lack of money	7 (4.2)
Previous experience of treating similar illness	101 (60.8)
Traditional belief	4 (2.4)
Unavailability of doctors	2 (1.2)
Others	6 (3.6)
Feeling after self-medication	
Temporary pain relief	86 (51.8)
Effective	52 (31.3)
Useful in stressful condition	16 (9.6)
Unsure about effects	5 (3.0)
Curative in nature	6 (3.6)
Cheaper action	1 (0.6)

Analgesics are the most widely used drugs by 120 (72.3%) of the study participants and the purchase is mostly done in pharmacy shops by 122 (73.5%) (Table 4).

**Table 4:** Types, sources, and consultation for self-medication and measures taken if the problem persists.

Questions	Frequency n (%)
Types of self-medication	
Analgesics	120 (72.3)
Antibiotics	16 (9.6)
Native herbs	16 (9.6)
Others	14 (8.4)
Purchase of medicines from	
Pharmacy shop	122 (73.5)
Hospital pharmacy	37 (22.3)
Traditional home	7 (4.2)
Advice is given by	
Relatives	35 (21.1)
Friends	23 (13.9)
Personal knowledge	71 (42.8)
Pharmacist	30 (18.1)
Mass media	7 (4.2)
If problem persists	
Visit a dentist	119 (71.7)
Visit a medical practitioner	39 (23.5)
Continue with the same medicine	8 (4.8)

**Table 5:** Socio-demographic factors and self-medication practice.

Characteristics	Prevalence of self-medication (n)
Gender	
Male	75
Female	91
Marital status	
Unmarried	73
Married	93
Education	
Illiterate	7
Up to secondary level	23
Higher secondary level	53
Bachelors	69
Masters	14
Age group	
18-24 years	63
25-34 years	58
35-44 years	20
45-54 years	16
>55 years	9
Distance from the nearest health center	
<1 km	84
1-2 km	38
>2 km	44

## DISCUSSION

In the current study, we assessed the self-medication practice among the patients visiting a dental hospital in Kathmandu. The age of the study participants ranged from 18-74 years with the mean age of 30.46 years. The majority of the participants were middle-aged and the prevalence of self-medication was found to be greater among them. This finding is following the study conducted by Shankar et al,^10^ and Komal Raj et al.^9^ The reason for this could be because the people of this age-group have knowledge and accessibility to various sources of information and also more workload and increased stress thereby lesser time to visit a dentist for their treatment needs.

Regarding the duration of self-medication, it was found that majority of the participants i.e. 59.6% had self-medication practice for few days which was similar to study by Komal Raj et al.^9^ Triggering factor for self-medication was found to be toothache in 60.8% participants in our study similar to study by Komal Raj et al.^9^ However it was not similar to study by Kalyan et al,^11^ in whom halitosis was found to be more common. The type of medication which was used in most of the participants in our study was analgesics in accordance to study by Kalyan et al,^11^ and Komal Raj et al,^9^ and not similar to studies by Mensah et al,^12^ where the majority of them i.e. 32.1% use antibiotics and Badiger et al,^13^ where the majority of them use antipyretics during self-medication. Our current study shows participants opting for self-medication practice due to previous experience of treating similar illness which is contrary the to the study by Komal Raj et al,^9^ in which the majority of the participants took advice from the pharmacist.

The prevalence of self-medication practice was found to be 62.6% in our study which is in accordance to study by Baig et al,^14^ which was found to be 57.3% and more compared to study by Kassie et al,^2^ where prevalence was 35.9%. The study by Komal Raj et al,^9^ showed a very high prevalence of self-medication practice i.e. 100% which may be due to various reasons like distance from the nearest health center, time factor or the belief in the clinical efficacy of traditional herbs. The majority of the participants (73.5%) purchase the medicine from the pharmacy based on their knowledge (42.8%) followed by instructions from relatives and pharmacists. The present study shows that there is a growing trend of self-medication practice. Owing to the various adverse reactions and side effects, the supply of medicines without a valid prescription should be prohibited.

## CONCLUSIONS

The prevalence of self-medication was found to be low as compared to the study done in similar settings. Self-medication practice is a sensitive issue that hasn't been given the required consideration. It is of concern to find every other dental patient being indulged in self-medication whether in the present or the past. It is the responsibility of the dental practitioner to create awareness among their patients about the various hazards of self-medication.

This can be done on an individual level as well as through various professional organizations. Also, proper control measures of dissemination of medicines and rational restriction of the availability of medicines to the public without prescription should be promoted.
